# Systematic evaluation of 2′-Fluoro modified chimeric antisense oligonucleotide-mediated exon skipping *in vitro*

**DOI:** 10.1038/s41598-019-42523-0

**Published:** 2019-04-15

**Authors:** Suxiang Chen, Bao T. Le, Madhuri Chakravarthy, Tamer R. Kosbar, Rakesh N. Veedu

**Affiliations:** 1 Centre for Molecular Medicine and Innovative Therapeutics, Murdoch University, Perth, 6150 Australia; 2 Perron Institute for Neurological and Translational Science, Perth, 6150 Australia

**Keywords:** DNA, Chemical modification, Nucleic acids

## Abstract

Antisense oligonucleotide (AO)-mediated splice modulation has been established as a therapeutic approach for tackling genetic diseases. Recently, Exondys51, a drug that aims to correct splicing defects in the dystrophin gene was approved by the US Food and Drug Administration (FDA) for the treatment of Duchenne muscular dystrophy (DMD). However, Exondys51 has relied on phosphorodiamidate morpholino oligomer (PMO) chemistry which poses challenges in the cost of production and compatibility with conventional oligonucleotide synthesis procedures. One approach to overcome this problem is to construct the AO with alternative nucleic acid chemistries using solid-phase oligonucleotide synthesis via standard phosphoramidite chemistry. 2′-Fluoro (2′-F) is a potent RNA analogue that possesses high RNA binding affinity and resistance to nuclease degradation with good safety profile, and an approved drug Macugen containing 2′-F-modified pyrimidines was approved for the treatment of age-related macular degeneration (AMD). In the present study, we investigated the scope of 2′-F nucleotides to construct mixmer and gapmer exon skipping AOs with either 2′-*O*-methyl (2′-*O*Me) or locked nucleic acid (LNA) nucleotides on a phosphorothioate (PS) backbone, and evaluated their efficacy in inducing exon-skipping in *mdx* mouse myotubes *in vitro*. Our results showed that all AOs containing 2′-F nucleotides induced efficient exon-23 skipping, with LNA/2′-F chimeras achieving better efficiency than the AOs without LNA modification. In addition, LNA/2′-F chimeric AOs demonstrated higher exonuclease stability and lower cytotoxicity than the 2′-*O*Me/2′-F chimeras. Overall, our findings certainly expand the scope of constructing 2′-F modified AOs in splice modulation by incorporating 2′-*O*Me and LNA modifications.

## Introduction

Nucleic acid therapeutics has attracted tremendous attention in recent years with a number of successful clinical translations for various diseases^1^. To date, the US Food and Drug Administration (FDA) has approved six oligonucleotide-based therapeutic molecules including one aptamer (Macugen) for the treatment of age-related macular degeneration (AMD), four antisense oligonucleotides (AOs) (Vitravene, Kynamro, Exondys51, and Spinraza) for the treatment of cytomegalovirus retinitis, familial hypercholesterolemia, Duchenne muscular dystrophy (DMD), and spinal muscular atrophy (SMA), respectively^2–4^ and one siRNA drug (Onpattro) for the treatment of amyloidosis. Unlike other protein-targeting therapeutic approaches, AOs can alter the pathological hallmark of the disease at the RNA level via different intracellular mechanisms (RNase-H-mediated degradation, imposing steric block to block translation or to modulate splicing)^1,5^. However, oligonucleotides composed of naturally occurring nucleotide monomers (deoxyribonucleotide or ribonucleotide) are easily degraded by nucleases and possess poor target binding affinity^5^, thus, they are not suitable for drug development. To overcome these limitations, chemically-modified nucleic acid analogues, mainly of sugar and phosphate backbone modifications, have been utilized in developing therapeutic oligonucleotides. So far, prominent chemical modifications that have been granted approval for clinical usage include phosphorothioate (PS)^6^, used in Vitravene, Kynamro, and Spinraza; 2′-*O*-methyl (2′-*O*Me)^7–11^, used in Macugen and Onpattro; 2′-Fluoro (2′-F)^7–11^, used in Macugen; 2′-*O*-methoxyethyl (2′-*O*MOE)^12^, used in Kynamro and Spinraza; and phosphorodiamidate morpholino (PMO)^13^, used in Exondys51. Of these, 2′-*O*Me and PMO have been explored extensively for AO-mediated splice modulation. In addition, several other analogues such as locked nucleic acid (LNA)^14–16^, unlocked nucleic acid (UNA)^17^, peptide nucleic acid (PNA)^18^, serinol nucleic acid (SNA)^19^, tricyclo DNA (tcDNA)^20^, twisted intercalating nucleic acid (TINA)^21^, anhydrohexitol nucleic acid (HNA)^22^, cyclohexenyl nucleic acid (CeNA)^22^, D-altritol nucleic acid (ANA)^22^ and morpholino nucleic acid (MNA)^23^ have also been investigated in splice modulation. Recently, nucleobase-modified AOs containing 2-thioribothymidine^24^, and 5-(phenyltriazol)-2′-deoxyuridine^25^ nucleotides have been reported to induce exon skipping in DMD model systems.

DMD is a severe and fatal muscle wasting genetic disorder mainly affecting newborn boys^26–28^. DMD is caused by one or more mutations in the dystrophin gene that ablate the expression of functional dystrophin proteins required for protecting muscle fibers from eccentric contraction and movement^29,30^. Recently, AO-mediated exon skipping has been established as one of the most promising therapeutic strategy for treating DMD^28,31–36^. Skipping the mutation containing exons can restore the dystrophin reading frame and rescue the production of the truncated but partially functional dystrophin protein. PMO and 2′-*O*Me-PS-modified AOs have been investigated in phase-3 clinical trials for DMD^27,28,32–38^. In 2016, the PMO-based AO drug (Exondys51) has been granted accelerated approval by the US FDA^3^. In contrast, the 2′-*O*Me-PS-based candidate (drisapersen) was rejected mainly due to safety issues and lack of efficacy^39^. Although PMO-modified oligonucleotides showed excellent safety profile, it is not compatible with standard oligonucleotide synthesis chemistries in order to synthesise as mixmers with other well-known nucleotide analogues and large-scale production of PMOs is challenging due to distinctive synthesis procedure. Therefore, it is necessary to evaluate alternative nucleic acid chemistries that can be used for AO drug development.

**Figure 1:**
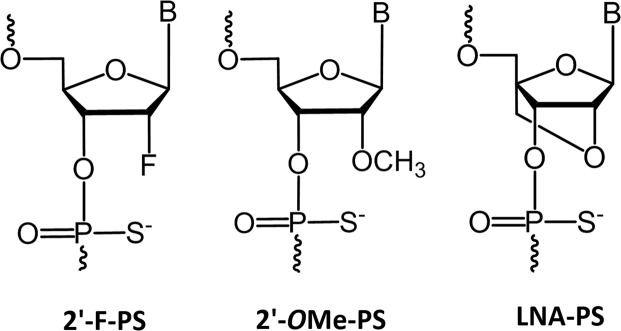
Structural representations of 2′-F, 2′-*O*Me, and LNA monomers on a PS backbone.

Towards this, Kawasaki *et al*. introduced 2′-F as an attractive ribonucleotide analogue for constructing AO. In fact, 2′-F-PS (Fig. 1) AOs showed higher target binding affinity, and nuclease stability^8^. Previous studies have also revealed its enhanced capability of inducing exon skipping *in vitro* compared to 2′-*O*Me-PS (Fig. 1) AOs^8–11^, which may be due to the recruitment of interleukin enhancer binding factors 2 and 3 (ILF2/3) by 2′-F AO/pre-mRNA duplex which may result in improved steric block efficiency^9–11^. However, 2′-F-modified AOs did not reach clinical evaluation and the scope of 2′-F-modified AOs needs to be improved by novel design approaches. LNA (Fig. 1) is another prominent RNA analogue that has been successfully investigated in recent studies to induce exon skipping in the dystrophin gene transcript^16,40^. In this study, for the first time, we herein report the design, synthesis and evaluation of 2′-F-modified exon skipping AOs to induce exon-23 skipping in DMD mouse myotubes *in vitro*.

## Results

In the present study, we used a previously reported fully modified 2′-*O*Me-PS 18-mer AO sequence, DmdE23D (+1–17) which was designed to induce exon-23 skipping in mouse *Dmd* transcript^16^, as a positive control (Table 1). Based on this AO, we systematically designed and synthesised a fully modified 2′-F AO on a PS backbone, three 2′-*O*Me/2′-F-PS chimeric AOs, and three LNA/2′-F-PS chimeric AOs which include a gapmer and two mixmer designs (Table 1). Two-step systematic evaluation was performed *in vitro* in mouse myotubes differentiated from H-*2K*^*b*^-tsA58 (H2K) *mdx* myoblasts. Initial evaluation was conducted for all AOs at 12.5 nM, 25 nM, and 50 nM concentrations while secondary evaluation was performed at lower concentrations (2.5 nM, 5 nM, and 12.5 nM) for chimeric AOs. In general, *H2K mdx* myoblasts were plated on 24-well plates and incubated for 24 h for differentiation. The differentiated myotubes were then transfected with different concentrations of the above-mentioned AOs by Lipofectin transfection reagent using a ratio of 2:1 (Lipofectin: AO). Twenty-four hours after transfection, cells were collected followed by total cellular RNA extraction, and reverse transcription polymerase chain reaction (RT-PCR) to amplify the dystrophin transcripts across exons 20–26 as reported previously^41^. Next, 2% agarose gel electrophoresis and densitometry (using Image J software) were performed to quantify the PCR products. The actual percentages of full length (901 bp), exon-23 skipping (688 bp), and exon-22/23 dual skipping (542 bp) products are presented based on the total amount of the dystrophin transcripts. Systematic exon skipping evaluation was performed in duplicates.

**Table 1:** List of AO names and sequences used in this study.

AO name	Sequence, 5′ → 3′ direction
Fully 2′-*O*Me-PS	**GCCAAACCUCGGCUUACC**
Fully 2′-F-PS	G^F^C^F^C^F^A^F^A^F^A^F^C^F^C^F^U^F^C^F^G^F^G^F^C^F^U^F^U^F^A^F^C^F^C^F^
2′-*O*Me/2′-F-PS gapmer	**GC**C^F^A^F^A^F^A^F^C^F^C^F^U^F^C^F^G^F^G^F^C^F^U^F^U^F^A^F^**CC**
2′-*O*Me/2′-F-PS mixmer 1	**G**C^F^C^F^A^F^A^F^A^F^**C**C^F^U^F^C^F^G^F^**G**C^F^U^F^U^F^A^F^C^F^**C**
2′-*O*Me/2′-F-PS mixmer 2	G^F^C^F^C^F^A^F^A^F^A^F^C^F^C^F^**U**C^F^G^F^**G**C^F^U^F^**U**A^F^C^F^**C**
LNA/2′-F-PS gapmer	***G***^**L**^**C**^**L**^C^F^A^F^A^F^A^F^C^F^C^F^U^F^C^F^G^F^G^F^C^F^U^F^U^F^A^F^***C***^**L**^***C***^**L**^
LNA/2′-F-PS mixmer 1	***G***^**L**^C^F^C^F^A^F^A^F^A^F^***C***^**L**^C^F^U^F^C^F^G^F^***G***^**L**^C^F^U^F^U^F^A^F^C^F^***C***^**L**^
LNA/2′-F-PS mixmer 2	G^F^C^F^C^F^A^F^A^F^A^F^C^F^C^F^***T***^L^C^F^G^F^***G***^**L**^C^F^U^F^***T***^**L**^A^F^C^F^***C***^**L**^

2′-*O*Me nucleotides are represented in underlined and bold letters; 2′-F nucleotides are represented in black letters with superscript F; LNA nucleotides are represented in underlined and bold-italic letters with superscript L. All AOs possess PS backbone.

### Evaluation of 2′-F modified AOs to induce exon skipping in dystrophin transcript in H2K *mdx* mouse myotubes *in vitro* at 12.5 nM, 25 nM, and 50 nM concentrations

**Figure 2:**
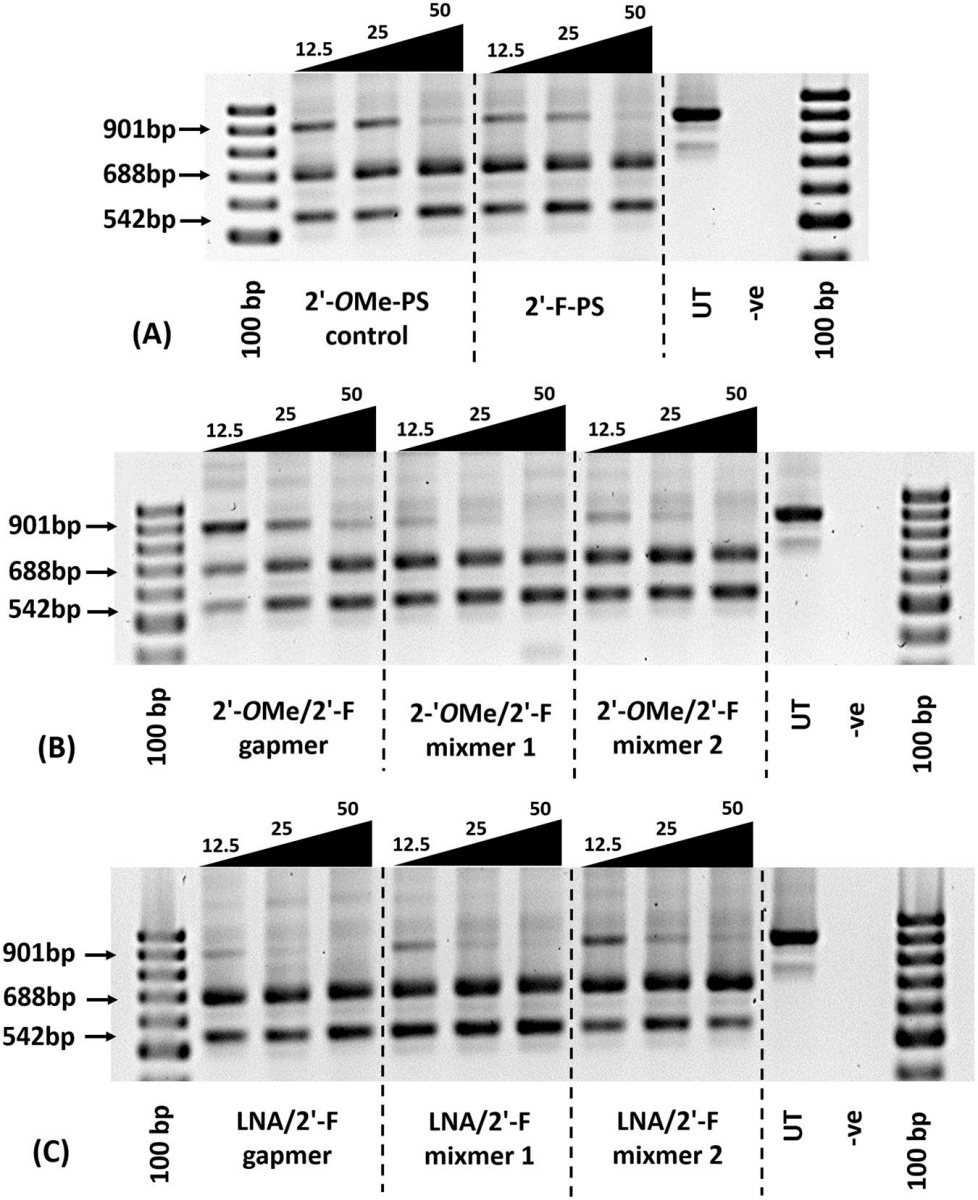
Agarose gel analysis of RT-PCR products showed exon-23 and exon-22/23 dual skipping in *mdx* mouse myotubes *in vitro*. Concentrations of AOs used include 12.5 nM, 25 nM, and 50 nM. (**A**) Fully modified 2′-*O*Me-PS control AO and fully modified 2′-F-PS AO; the original gel image is shown in Fig. S1 (A2) (Supplementary Information). (**B**) 2′-*O*Me modified 2′-F-PS AO chimeras including 2′-*O*Me/2′-F-PS gapmer, 2′-*O*Me/2′-F-PS mixmer 1, and 2′-*O*Me/2′-F-PS mixmer 2; the original gel image is shown in Fig. S1(B2) (Supplementary Information). (**C**) LNA modified 2′-F-PS AO chimeras including LNA/2′-F-PS gapmer, LNA/2′-F-PS mixmer 1, and LNA/2′-F-PS mixmer 2; the original gel image is shown in Fig. S1(C2) (Supplementary Information). The corresponding densitometry data of the gel images are shown in Fig. S1(A1, B1 and C1) (Supplementary Information).

**Figure 3:**
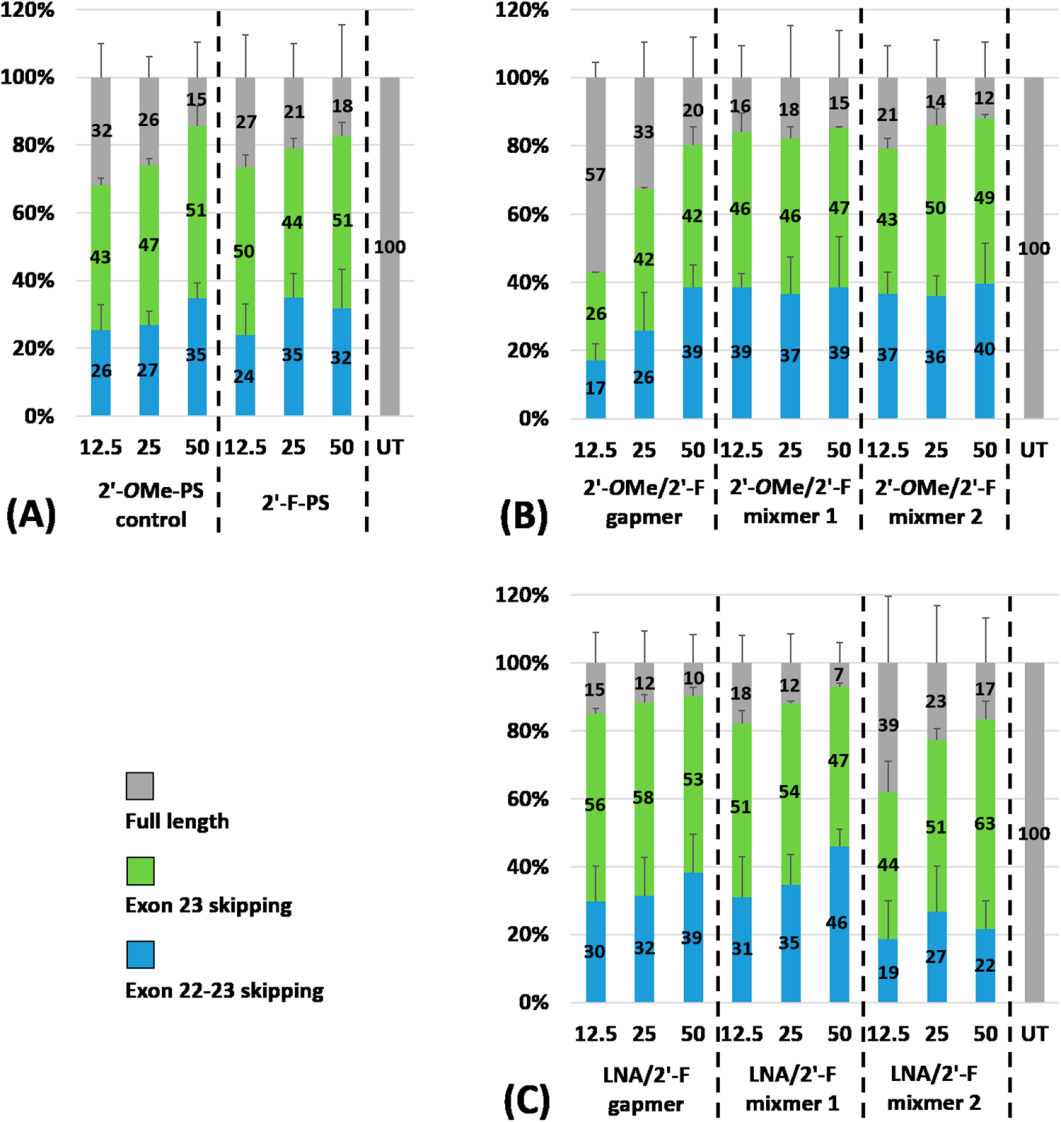
Densitometry analysis of RT-PCR products (in duplicates) showed exon-23 and exon-22/23 dual skipping in *mdx* mouse myotubes *in vitro*. Concentrations of AOs used include 12.5 nM, 25 nM, and 50 nM. (**A**) Fully modified 2′-*O*Me-PS control AO and fully modified 2′-F-PS AO; (**B**) 2′-*O*Me modified 2′-F-PS AO chimeras including 2′-*O*Me/2′-F-PS gapmer, 2′-*O*Me/2′-F-PS mixmer 1, and 2′-*O*Me/2′-F-PS mixmer 2; (**C**) LNA modified 2′-F-PS AO chimeras including LNA/2′-F-PS gapmer, LNA/2′-F-PS mixmer 1, and LNA/2′-F-PS mixmer 2. The gel images and their corresponding densitometry data of each repetition are shown in Figs S1 and S3 (Supplementary Information).

Firstly, we evaluated the exon skipping efficiency of all AOs (Table 1) *in vitro* at three different concentrations (12.5 nM, 25 nM, and 50 nM). The results demonstrated that all AOs are capable of inducing efficient exon skipping at various levels (Figs 2 and 3). In line with previous report^16^, the 2′-*O*Me-PS control AO induced efficient exon-23 skipping by yielding the skipped product of 688 bp at all concentrations (43% at 12.5 nM, 47% at 25 nM, and 51% at 50 nM; Fig. 3A). Interestingly, the fully modified 2′-F-PS AO showed higher exon-23 skipping at 12.5 nM (50%) compared to the control AO (43%), but the efficiency reduced to 44% at 25 nM and remained 51% at 50 nM, respectively. But, 2′-F-PS AOs showed the undesired exon-22/23 dual skipping product of 542 bp in higher yield at 25 nM (35%) compared to 2′-*O*Me-PS AO (27%) (Fig. 3A).

All mixmer and gapmer AOs achieved the highest exon-23 skipping at 25 nM or 50 nM (Fig. 3B,C). Markedly, the LNA/2′-F-PS mixmer 2 yielded the highest exon-23 skipping product (63%) (Fig. 3C) compared to all other AOs (42–53%) at 50 nM concentration (Fig. 3). Surprisingly, a significant drop in exon-23 skipping product was observed after transfection with 12.5 nM of the 2′-*O*Me/2′-F-PS gapmer, which induced only 26% (Fig. 3B) of skipping while other AOs achieved 43–56% of skipping at this concentration (Fig. 3). Direct comparison between the 2′-*O*Me/2′-F-PS and the corresponding LNA/2′-F-PS gapmer and mixmer AOs indicated that the LNA/2′-F-PS AOs achieved higher exon-23 skipping efficiency at various concentrations. For instance, at 25 nM concentration, exon-23 skipping induced by LNA/2′-F gapmer (58%) was higher than 2′-*O*Me/2′-F gapmer (42%), LNA/2′-F mixmer 1 (54%) was higher than 2′-*O*Me/2′-F mixmer 1 (46%), and LNA/2′-F mixmer 2 (51%) was higher than 2′-*O*Me/2′-F mixmer 2 (50%). Furthermore, at all concentrations, each type of LNA/2′-F mixmers (mixmer 1: 31% at 12.5 nM, 35% at 25 nM; mixmer 2: 19% at 12.5 nM, 27% at 25 nM, 22% at 50 nM) induced the less undesired exon-22/23 dual skipping than its corresponding 2′-*O*Me/2′-F mixmers (mixmer 1: 39% at 12.5 nM, 37% at 25 nM; mixmer 2: 37% at 12.5 nM, 36% at 25 nM, 40% at 50 nM) except mixmer 1 AOs at 50 nM (LNA/2′-F mixmer 1: 46%; 2′-*O*Me/2′-F mixmer 1: 39%).

### Evaluation of chimeric AOs to induce exon skipping in dystrophin transcript in H2K *mdx* mouse myotubes *in vitro* at 2.5 nM, 5 nM, and 12.5 nM concentrations

**Figure 4:**
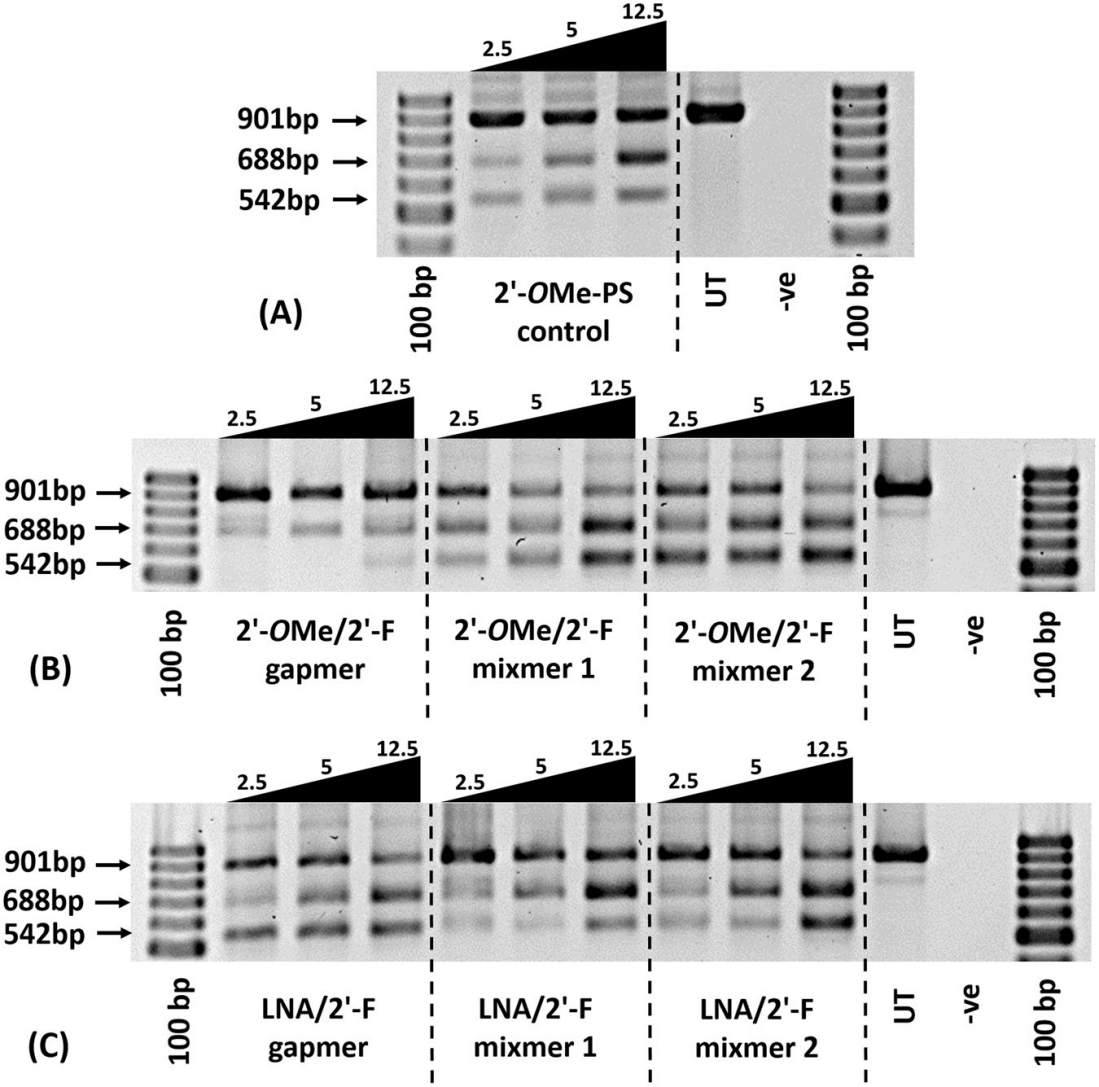
Agarose gel analysis of the RT-PCR products showed exon-23 and exon-22/23 dual skipping in *mdx* mouse myotubes *in vitro*. Concentrations of AOs used include 2.5 nM, 5 nM, and 12.5 nM. (**A**) Fully modified 2′-*O*Me-PS control AO; the original gel image is shown in Fig. S2(A2) (Supplementary Information). (**B**) 2′-*O*Me modified 2′-F-PS AO chimeras including 2′-*O*Me/2′-F-PS gapmer, 2′-*O*Me/2′-F-PS mixmer 1, and 2′-*O*Me/2′-F-PS mixmer 2; the original gel image is shown in Fig. S2(B2) (Supplementary Information). (**C**) LNA modified 2′-F-PS AO chimeras including LNA/2′-F-PS gapmer, LNA/2′-F-PS mixmer 1, and LNA/2′-F-PS mixmer 2; the original gel image is shown in Fig. S2(C2) (Supplementary Information). The corresponding densitometry data of the gel images are shown in Fig. S2(A1,B1,C1) (Supplementary Information).

**Figure 5:**
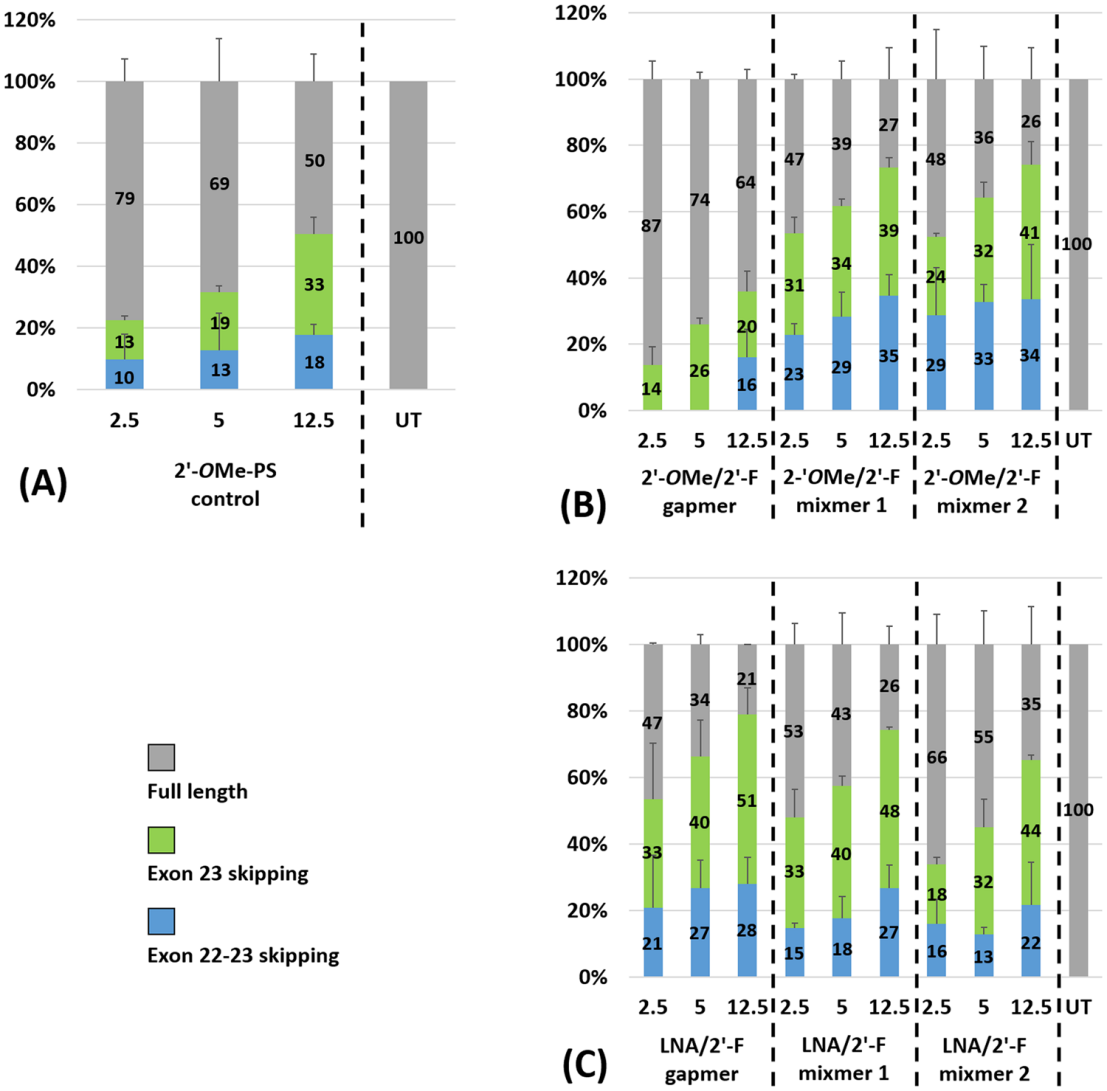
Agarose gel and densitometry analysis of the RT-PCR products (in duplicates) showed exon-23 and exon-22/23 dual skipping in *mdx* mouse myotubes *in vitro*. Concentrations of AOs used include 2.5 nM, 5 nM, and 12.5 nM. (A) Fully modified 2′-*O*Me-PS control AO; (B) 2′-*O*Me modified 2′-F-PS AO chimeras including 2′-*O*Me/2′-F-PS gapmer, 2′-*O*Me/2′-F-PS mixmer 1, and 2′-*O*Me/2′-F-PS mixmer 2; (C) LNA modified 2′-F-PS AO chimeras including LNA/2′-F-PS gapmer, LNA/2′-F-PS mixmer 1, and LNA/2′-F-PS mixmer 2. The gel images and their corresponding densitometry data of each repetition are shown in Figs S2 and S4 (Supplementary Information).

To further explore the ability of the 2′-F modified chimeric AOs in inducing exon skipping, we transfected all chimeric AOs (2′-*O*Me/2′-F-PS chimeras and LNA/2′-F-PS chimeras) (Table 1) at lower concentrations (2.5 nM, 5 nM, and 12.5 nM) together with 2′-*O*Me-PS control. In general, all AOs yielded efficient exon-23 skipped products in a dose-dependent manner, except the 2′-*O*Me/2′-F gapmer (Figs 4 and 5). Furthermore, all 2′-F modified chimeras achieved higher exon-23 skipping efficiency than the 2′-*O*Me-PS control at all concentrations except 2′-*O*Me/2′-F gapmer at 12.5 nM (2′-*O*Me/2′-F gapmer: 20%; 2′-*O*Me-PS control: 33%) (Fig. 5). Notably, at 12.5 nM, LNA/2′-F-PS gapmer achieved the highest exon-23 skipping level (51%) (Fig. 5C) in comparison to all other chimeric AOs (20–48%) (Fig. 5B,C). It was also noted that LNA/2′-F chimeric AOs achieved higher or comparable exon-23 skipping efficiency compared with its corresponding 2′-*O*Me/2′-F chimeric AO at all concentrations except LNA/2′-F mixmer 2 at 2.5 nM (LNA/2′-F mixmer 2: 18%; 2′-*O*Me/2′-F mixmer 2: 24%) (Fig. 5B,C). The percentage of exon-23 skipping induced by LNA/2′-F gapmer (33% at 2.5 nM, 40% at 5 nM, 51% at 12.5 nM) was higher than 2′-*O*Me/2′-F gapmer (14% at 2.5 nM, 26% at 5 nM, 20% at 12.5 nM); LNA/2′-F mixmer 1 (33% at 2.5 nM, 40% at 5 nM, 48% at 12.5 nM) was higher than 2′-*O*Me/2′-F mixmer 1 (31% at 2.5 nM, 34% at 5 nM, 39% at 12.5 nM); and LNA/2′-F mixmer 2 (32% at 5 nM, 44% at 12.5 nM) was higher than or equivalent to 2′-*O*Me/2′-F mixmer 2 (32% at 5 nM, 41% at 12.5 nM). In addition, at all concentrations (2.5 nM, 5 nM, 12.5 nM), LNA/2′-F mixmers (mixmer 1: 15%, 18%, 27%; mixmer 2: 16%, 13%, 22%) induced less undesired exon-22/23 dual skipping than their corresponding 2′-*O*Me/2′-F mixmer (mixmer 1: 23%, 29%, 35%; mixmer 2: 29%, 33%, 34%) (Fig. 5B,C). Interestingly, exon-22/23 dual skipping was not visible in the case of 2′-*O*Me/2′-F-PS gapmer at 2.5 nM and 5 nM (Figs 4B and 5B).

To further evaluate the optimal design of the AO, we compared the exon skipping efficiency between the gapmer and two mixmer chimeric AOs. The results demonstrated that at all concentrations (2.5 nM, 5 nM, 12.5 nM), the 2′-*O*Me/2′-F mixmer chimeras achieved higher exon-23 skipping efficiency than the 2′-*O*Me/2′-F gapmer chimeras (Figs 4B and 5B), however, the LNA/2′-F gapmer induced higher or comparable exon-23 skipping compared with the LNA/2′-F mixmers. That is, exon-23 skipping induced by 2′-*O*Me/2′-F mixmers (mixmer 1: 31% at 2.5 nM, 34% at 5 nM, 39% at 12.5 nM; mixmer 2: 24% at 2.5 nM, 32% at 5 nM, 41% at 12.5 nM) was higher than 2′-*O*Me/2′-F gapmer (14% at 2.5 nM, 26% at 5 nM, 20% at 12.5 nM) (Fig. 5B); while exon-23 skipping induced by LNA/2′-F gapmer (33% at 2.5 nM, 40% at 5 nM, 51% at 12.5 nM) was higher than or comparable to the LNA/2′-F mixmers (mixmer 1: 33% at 2.5 nM, 40% at 5 nM, 48% at 12.5 nM; mixmer 2: 18% at 2.5 nM, 32% at 5 nM, 44% at 12.5 nM) (Fig. 5C). On the other hand, LNA/2′-F mixmers induced less unwanted exon-22/23 dual skipping than LNA/2′-F gapmer. Therefore, exon-22/23 skipping induced by LNA/2′-F mixmers (mixmer 1: 15% at 2.5 nM, 18% at 5 nM, 27% at 12.5 nM; mixmer 2: 16% at 2.5 nM, 13% at 5 nM, 22% at 12.5 nM) was lower than LNA/2′-F gapmer (21% at 2.5 nM, 27% at 5 nM, 28% at 12.5 nM) (Fig. 5C).

### Evaluation of *in vitro* cytotoxicity of the 2′-F modified AOs

**Figure 6:**
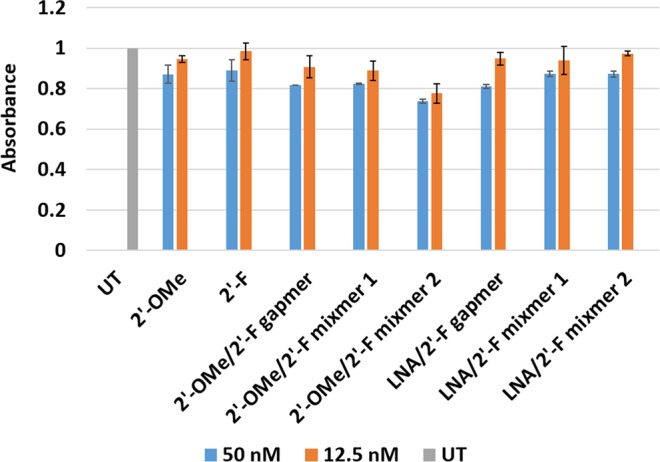
Cell viability assay (in duplicates) of 2′-F modified AOs. The original data of absorbance is shown in Table S1 (Supplementary Information).

Safety is crucial for any clinically relevant therapeutic drug. Therefore, we performed the cytotoxicity evaluation for all 2′-F modified AOs by conducting WST-1 reagent-based cell viability assay. Briefly, mouse myoblasts were seeded and differentiated into myotubes, followed by transfecting with the AOs (50 nM and 12.5 nM) as described previously. The untreated (UT) groups were not transfected by any AO but only incubated with Lipofectin reagent instead. The cells were then incubated with WST-1 reagent at a ratio of 1:10 (v/v) at 37 °C, 5% CO_2_ for 4 h. Cytotoxicity was determined by measuring the absorbance at 450 nm. In general, all 2′-F modified AOs did not show any significant cytotoxicity in comparison to the fully 2′-*O*Me-PS control (Fig. 6). Notably, at 12.5 nM, LNA modified 2′-F chimeras showed higher viability than 2′-*O*Me modified 2′-F chimeras. On the other hand, 2′-*O*Me/2′-F-PS mixmer 2 demonstrated less viability than the other AOs at both concentrations, which may be due to the positioning of the 2′-*O*Me nucleotides as the 2′-*O*Me/2′-F-PS mixmer 1 achieved higher cell viability at both concentrations (Fig. 6).

### Evaluation of *in vitro* nuclease stability of the 2′-F modified AOs

**Figure 7:**
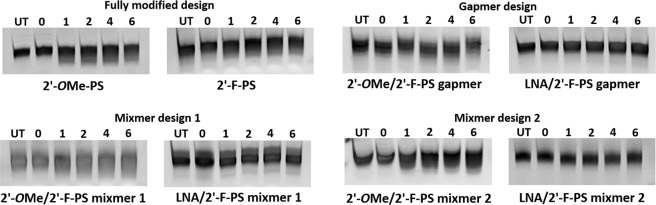
Nuclease stability analysis of 2′-F modified AOs. The original gel images are shown in Fig. S5 (Supplementary Information).

To gain more insight into the AO stability, we then performed the nuclease degradation assay of all the 2′-F modified AOs in comparison to the fully 2′-*O*Me-PS control. In short, AOs were incubated with Phosphodiesterase I from Crotalus adamanteus venom which possesses very high exonuclease activity, at 37 °C for various incubation periods including 0, 1, 2, 4, and 6 h. Samples were collected at the desired timepoints and quenched with formamide loading buffer, followed by denaturation at 95 °C for 5 min. Next, 20% denaturing polyacrymide gel analysis was performed and the results were analysed by gel imaging. All 2′-F modified AOs demonstrated high stability under the applied conditions compared to the fully 2′-*O*Me-PS control (Fig. 7). Not surprisingly, all the LNA/2′-F-PS chimeras showed higher nuclease resistance than the other AOs without LNA modification (Fig. 7). In addition, the gapmer chimeras showed higher stability than the mixmer chimeras.

## Discussion

Therapeutic potential of AOs was first demonstrated by Zamecnik *et al*. in 1978^42^. Stemming from this initial work, AOs have been extensively explored as a potential gene-targeting approach for the treatment of various genetic diseases. In line with this, splice-switching AOs have been developed, firstly by Dominski *et al*. in 1993, and later became promising therapies towards tackling genetic diseases caused by mutations such as DMD and SMA^43^. Along this line, chemically-modified nucleic acid analogues play a crucial role in the successful clinical translation of any AO-based drug, given the PMO-based Exondys51 was granted conditional approval in 2016, while the 2′-*O*Me-PS-based drisapersen was rejected in the same year due to lack of efficacy and life-threatening side effects. Although a PMO-based AO is relatively non-toxic, PMO chemistry has its disadvantages due to limitation of large-scale synthesis and lack of compatibility with other chemistries. Towards exploring chemically modified AOs targeting DMD, Aartsma-Rus and colleagues evaluated AOs constructed by fully modified 2′-F-PS and LNA-PS^10,11,44^, while our group has investigated LNA, HNA, CeNA, ANA, and MNA monomers^16,22,23^.

The first attempt to study 2′-F modified AO was reported in 1993 when Kawasaki *et al*. found that 2′-F modification enhanced the AO’s target binding affinity to their complementary RNA compared to 2′-*O*Me modified AO, and showed excellent nuclease stability^8^. Two decades later, Rigo and colleagues discovered a unique property of the 2′-F modified AO/target pre-mRNA duplex that is able to recruit the ILF2/3 proteins, resulting in exon-7 skipping of *SMN2* mRNA in a SMA model system^9^. Based on this finding, Aartsma-Rus and coworkers compared the exon skipping capability of the fully 2′-F-PS and fully 2′-*O*Me-PS AOs in targeting DMD, and showed that 2′-F-PS AO induced higher human exon-53 and mouse exon-23 skipping *in vitro*^10,11^, which was not surprising as 2′-F modification has many advantages as proven by Kawasaki *et al*.^8^ and Rigo *et al*.^9^, however, 2′-F-PS AO was less efficient than 2′-*O*Me-PS *in vivo* and indicated toxicity in mice^11^. Thus, their results did not support clinical use of 2′-F-PS AOs^11^.

In an attempt to improve the therapeutic potential of 2′-F modified AO, we incorporated 2′-*O*Me-PS and LNA-PS nucleotides into an 18 mer 2′-F-PS AO sequence that contained either four 2′-*O*Me or LNA nucleotides designed to target exon-23 of *mdx* mouse myotubes (Table 1). The efficacies of the AOs were first evaluated at higher (12.5 nM, 25 nM, 50 nM), and then lower concentrations (2.5 nM, 5 nM, 12.5 nM); in addition to performing cytotoxicity and nuclease stability analysis.

Fully modified 2′-F-PS AO induced higher exon-23 skipping than the fully modified 2′-*O*Me-PS control at 12.5 nM, and both of them indicated similar exon-23 skipping efficiency on higher concentrations (25 nM, 50 nM), in line with Aartsma-Rus *et al*.^12^ finding that 2′-F-PS AO induced minimal increase of exon-23 skipping when compared to 2′-*O*Me-PS AO *in vitro*. In general, chimeric 2′-F-PS AOs induced efficient exon-23 skipping. Each of the LNA/2′-F-PS chimeras achieved higher or comparable exon-23 skipping in comparison to their corresponding 2′-*O*Me/2′-F-PS counterparts (LNA/2′-F-PS gapmer > 2′-*O*Me/2′-F-PS gapmer, LNA/2′-F-PS mixmer 1 ≥ 2′-*O*Me/2′-F-PS mixmer 1, LNA/2′-F-PS mixmer 2 > 2′-*O*Me/2′-F-PS mixmer 2), indicating that LNA modification may possess better exon-23 skipping capability than 2′-*O*Me on a 2′-F-PS platform. Notably, at 50 nM, LNA/2′-F-PS mixmer 2 achieved the highest exon-23 skipping over other AOs, and, 2′-*O*Me/2′-F-PS gapmer induced the least exon-23 skipping compared to the others at 12.5, 25, and 50 nM, suggesting that the positioning of LNA or 2′-*O*Me analogues also affects exon skipping efficiency. Interestingly, at 12.5 and 25 nM concentrations, the LNA/2′-F mixmers induced less undesired exon-22/23 dual skipping than their corresponding 2′-*O*Me/2′-F mixmers, suggesting that LNA/2′-F mixmers may be a better tool for exon skipping application compared to 2′-*O*Me/2′-F mixmers.

At lower concentrations (2.5 nM, 5 nM, 12.5 nM), all 2′-*O*Me/2′-F mixmer AOs showed higher exon-23 skipping than their gapmer counterpart at all three concentrations, while LNA/2′-F gapmer AO showed higher or comparable exon-23 skipping in comparison to its mixmer counterparts. This further suggests that the positioning of 2′-*O*Me or LNA nucleotides on a 2′-F-PS platform affects exon skipping efficiency. On the other hand, each of the LNA/2′-F-PS chimeras achieved higher or comparable exon-23 skipping compared with their corresponding 2′-*O*Me/2′-F-PS counterparts at 5 nM and 12.5 nM (LNA/2′-F-PS gapmer > 2′-*O*Me/2′-F-PS gapmer, LNA/2′-F-PS mixmer 1 > 2′-*O*Me/2′-F-PS mixmer 1, LNA/2′-F-PS mixmer 2 ≥ 2′-*O*Me/2′-F-PS mixmer 2), which is consistent with previous experiments, and confirmed that LNA modification can result in higher exon-23 skipping than 2′-*O*Me on a 2′-F-PS platform. Furthermore, similar to the observation at higher concentrations (12.5 nM, 25 nM, 50 nM), the LNA/2′-F mixmers induced less exon-22/23 dual skipping than its corresponding 2′-*O*Me/2′-F mixmers at all concentrations, which further confirmed that LNA/2′-F mixmers may be more preferable than 2′-*O*Me/2′-F mixmers in exon skipping application.

In addition, cell viability assay performed to assess the cytotoxicity of the AOs showed that all 2′-F modified chimeric AOs achieved comparable cytotoxicity profiles in comparison to their fully 2′-*O*Me-PS control and the fully modified 2′-F-PS AO. Notably, the LNA/2′-F-PS chimeras showed better safety profile than the 2′-*O*Me/2′-F-PS chimeras, and the 2′-*O*Me/2′-F-PS mixmer 2 appeared to be the most toxic AO compared to others. This suggests that nucleotide positioning can be important in optimizing AO toxicity, although further evaluation in *in vivo* models are required. Nuclease stability assay demonstrated that fully 2′-F-PS AO and 2′-*O*Me/2′-F-PS chimeras possess similar stability as their fully 2′-*O*Me-PS control *in vitro*. Not surprisingly, LNA/2′-F-PS chimeras were more stable than the other AOs without LNA modification.

In conclusion, 2′-F-PS modified AOs induce higher *Dmd* exon-23 skipping efficiency than fully 2′-*O*Me-PS AO. Introduction of LNA nucleotides into 2′-F-PS sequence further improved exon-23 skipping efficiency, while not compromising cell viability and nuclease stability, in comparison to the 2′-*O*Me/2′-F-PS chimeras. In addition, mixmer designs of 2′-*O*Me/2′-F chimeras achieved higher efficiency of exon-23 skipping than their gapmer counterpart, while gapmer design of LNA/2′-F chimeras achieved higher efficiency of exon-23 skipping than their mixmer counterparts. Collectively, our findings expand the scope of utilizing 2′-F modified AOs in splice modulation application by constructing 2′-*O*Me and LNA-modified 2′-F-PS chimeras. Specifically, we suggest that development of LNA modified 2′-F-PS mixmer or gapmer chimeric AOs, may present a promising therapeutic strategy for DMD.

## Methods

### Design and synthesis of chemically modified AOs

All AOs (Table 1) were synthesised in-house on an Expedite^TM^ 8909 oligonucleotide synthesiser (Applied Biosystems) via standard phosphoramidite chemistry at 1 µmol scale. Deprotection was performed by incubating the crude oligonucleotides with ammonium hydroxide (Sigma; cat#: 221228-500 ml) at 55 °C overnight, followed by desalting through illustra NAP-10 columns (GE Healthcare; cat#: 45-000-153). All synthesis reagents were purchased from Merck Millipore.

### Cell culture and transfection

*H-2K*^*b*^-tsA58 (H2K) *mdx* mouse myoblasts were cultured and differentiated as described previously^41,45,46^. Briefly, when 60–80% confluent, myoblast cultures were treated with trypsin (ThermoFisher Scientific; cat#: 15400054) and seeded on a 24-well plate at a density of 2 × 10^4^ cells/well. The plates were pre-treated with 50 μg/mL poly-D-lysine (Merck Millipore; cat#: P7886-50mg), followed by 100 μg/mL Matrigel (Corning Life Science; cat#: FAL354234). Myoblasts were differentiated into myotubes in Dulbecco’s Modified Eagle Medium (DMEM) (ThermoFisher Scientific; cat#: 11885084) containing 5% horse serum (ThermoFisher Scientific; cat#: 16050122) by incubating at 37 °C, 5% CO_2_ for 24 h. AOs were complexed with Lipofectin transfection reagent (ThermoFisher Scientific; cat#: 18292011) at the ratio of 2:1 (v:w) (Lipofectin:AO) and used in a final transfection volume of 500 μL/well in a 24-well plate as per the manufacturer’s instructions.

### RNA isolation and reverse transcription polymerase chain reaction (RT-PCR)

Total RNA was extracted from transfected mouse myotubes using Direct-zol™ RNA MiniPrep Plus with TRI Reagent^®^ (Zymo Research, supplied through Integrated Sciences; cat#: R2052) as per the manufacturer’s instructions. The dystrophin transcripts were then analysed by RT-PCR using SuperScript™ III Reverse Transcriptase III (ThermoFisher Scientific; cat#: 12574026) across exons-20 to 26 as described previously^38^. PCR products were separated on a 2% agarose gel in Tris–acetate–EDTA buffer and the images were captured on a Fusion Fx gel documentation system (Vilber Lourmat). Densitometry was performed by Image J software^47^. To quantify the actual exon skipping efficacy induced by AOs, the amount of full length (901 bp), exon-23 skipping (688 bp), and exon-22/23 dual skipping (542 bp) products are expressed as percentages of total dystrophin transcript products.

### *In vitro* cytotoxicity analysis of AOs

Myoblasts were seeded, allowed to differentiate to myotubes, and transfected with AOs at 50 and 12.5 nM as described previously. 24 h after transfection, cytotoxicity was determined by a colorimetric assay using WST-1 reagent (2-(4-iodophenyl)-3-(4-nitro-phenyl)-5-(2,4-disulfophenyl)-2*H*-tetrazolium) (Sigma; cat#: ab65473). Briefly, WST-1 solution was added at a ratio of 1:10 (v/v) per well and incubated for 4 h at 37 °C, 5% CO_2_. The absorbance was then measured in a microplate reader (FLUOstar Omega, BMG Labtech) at 450 nm.

### *In vitro* nuclease stability analysis of AOs

Stability of all the AOs (Table 1) against 3′ end to 5′ end exonuclease degradation was evaluated using 0.08 units/reaction of Phosphodiesterase I from Crotalus adamanteus venom (Sigma; cat#: P3134-100MG) in a buffer of 10 mM Tris-HCL, 100 mM NaCl, and 15 mM MgCl_2_ in a final volume of 45 μL. Briefly, samples were incubated at 37 °C and 7.5 μL of reaction aliquots were removed at different time points (0, 1, 2, 4, and 6 h) and an equal volume of 80% formamide containing bromophenol blue and xylene cyanol gel tracking dyes was then added, followed by heating for 5 min at 95 °C. Next, the products were separated on a 20% denaturing polyacrylamide gel. Quantitation was performed on a Fusion Fx gel documentation system (Vilber Lourmat).

## Supplementary information

Supplementary Information
