# Author Correction: Systematic evaluation of 2′-Fluoro modified chimeric antisense oligonucleotide-mediated exon skipping *in vitro*

**DOI:** 10.1038/s41598-020-63706-0

**Published:** 2020-04-16

**Authors:** Suxiang Chen, Bao T. Le, Madhuri Chakravarthy, Tamer R. Kosbar, Rakesh N. Veedu

**Affiliations:** 1 Centre for Molecular Medicine and Innovative Therapeutics, Murdoch University, Perth, 6150 Australia; 2 Perron Institute for Neurological and Translational Science, Perth, 6150 Australia

Correction to: *Scientific Reports* 10.1038/s41598-019-42523-0, published online 15 April 2019

The original version of this Article contained errors.

The author Tamer R Kosbar was incorrectly affiliated with “Perron Institute for Neurological and Translational Science, Perth, 6150, Australia”. The correct affiliation is listed below.

Centre for Molecular Medicine and Innovative Therapeutics, Murdoch University, Perth, 6150, Australia.

There was also an error in Figure 3C, where the x-axis labels “LNA/2’-F mixmer 1” and “LNA/2’-F mixmer 2” were incorrectly given as “2-‘*O*Me/2’-F mixmer 1” and “2-‘*O*Me/2’-F mixmer 2” respectively.

The Acknowledgements section contained a typographical error where,

“The authors thank Prof. Steve Wilton and Prof. Sue Fletcher for kindly providing H2K cells, Prithi Raguraman for assistance towards experiments, Yanying An and Jessica Cale for help towards figure preparation and Kristin West for informative discussion.”

was incorrectly given as:

“The authors thank Prithi Raguraman for assistance towards experiments, Yanying An and Jessica Cale for help towards figure preparation and Kristin West for informative discussion.”

In addition, the Supplementary Information file contained an error in Supplementary Figure S3, where the labels of gel C2 were shifted slightly to the right.

These errors have now been corrected in the PDF and HTML versions of the Article, as well as in the accompanying Supplementary Information file.

