# Supplementary material for: Systematic evaluation of 2′-Fluoro modified chimeric antisense oligonucleotide-mediated exon skipping in vitro
Source: Sci Rep. 2019 Apr 15;9:6078. doi: 10.1038/s41598-019-42523-0 (PMC6465270; doi:10.1038/s41598-019-42523-0)
Supplement: Supplementary file 1 — Supplementary Information [file 41598_2019_42523_MOESM1_ESM.docx]

**Supplementary Information**

**Systematic evaluation of 2'-Fluoro modified chimeric antisense oligonucleotide-mediated exon skipping *in vitro***

**Suxiang Chen** ^1,2^**, Bao T. Le** ^1,2^**, Madhuri Chakravarthy**^1,2^, **Tamer R. Kosbar** ^1^**, and Rakesh N. Veedu** ^1,2*^

^1^Centre for Molecular Medicine and Innovative Therapeutics, Murdoch University, Perth, Australia- 6150

^2^Perron Institute for Neurological and Translational Science, Perth, Australia- 6150

^*^Address for Correspondence: [R.Veedu@murdoch.edu.au](mailto:R.Veedu@murdoch.edu.au)


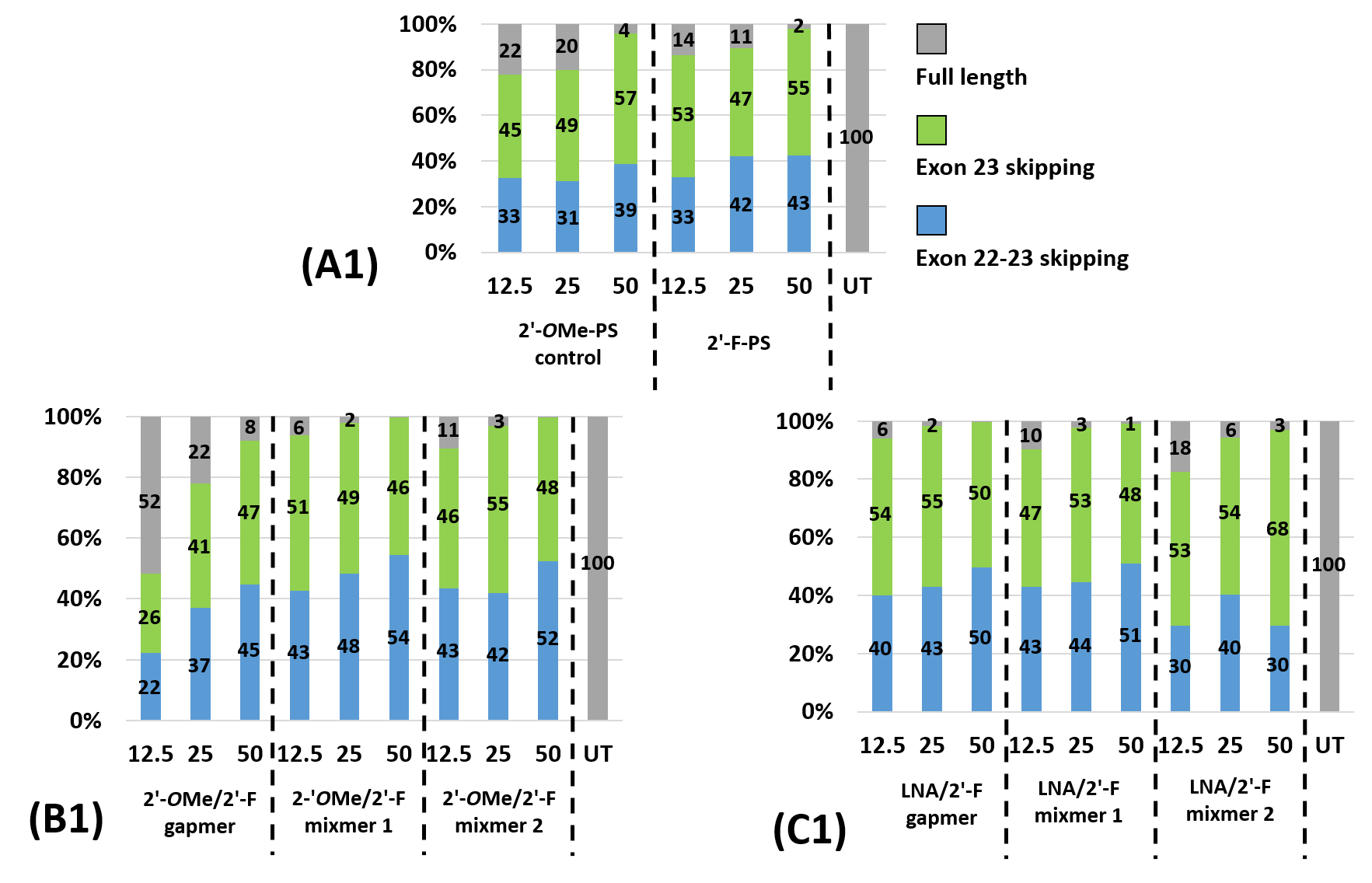


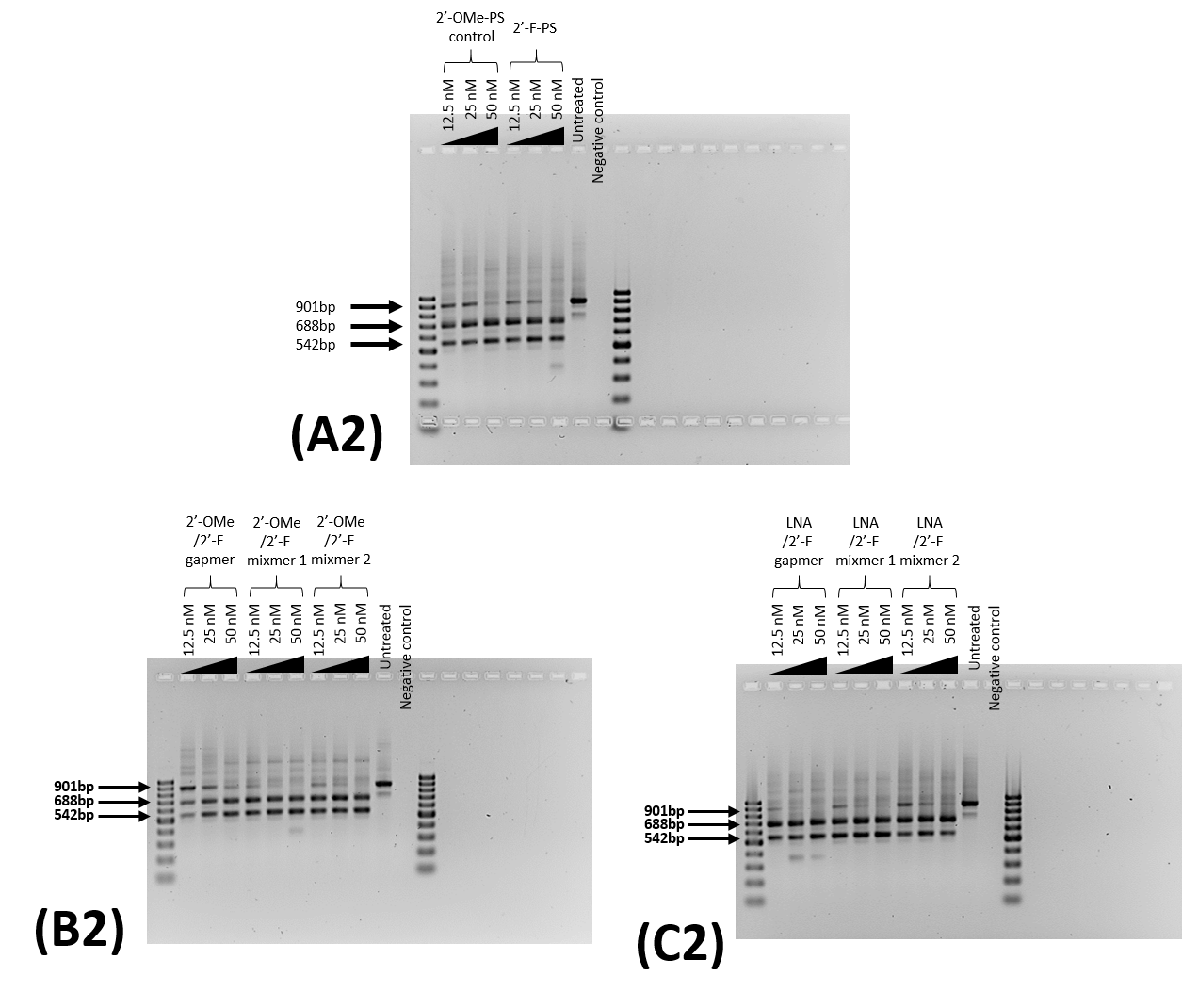


**Supplementary Figure S1**. Densitometry analysis and original gel images of repetition one of RT-PCR products showed exon-23 and exon-22/23 dual skipping in *mdx* mouse myotubes *in vitro*. Concentrations of AOs used include 12.5 nM, 25 nM, and 50 nM. (A1, A2) Fully modified 2'-*O*Me-PS control AO and fully modified 2'-F-PS AO; (B1, B2) 2'-*O*Me modified 2'-F-PS AO chimeras including 2'-*O*Me/2'-F-PS gapmer, 2'-*O*Me/2'-F-PS mixmer 1, and 2'-*O*Me/2'-F-PS mixmer 2; (C1, C2) LNA modified 2'-F-PS AO chimeras including LNA/2'-F-PS gapmer, LNA/2'-F-PS mixmer 1, and LNA/2'-F-PS mixmer 2. [The original gels and densitometry data in this figure represent and/or support the gel images in Figure 2 and densitometry analysis in Figure 3 of the paper.]


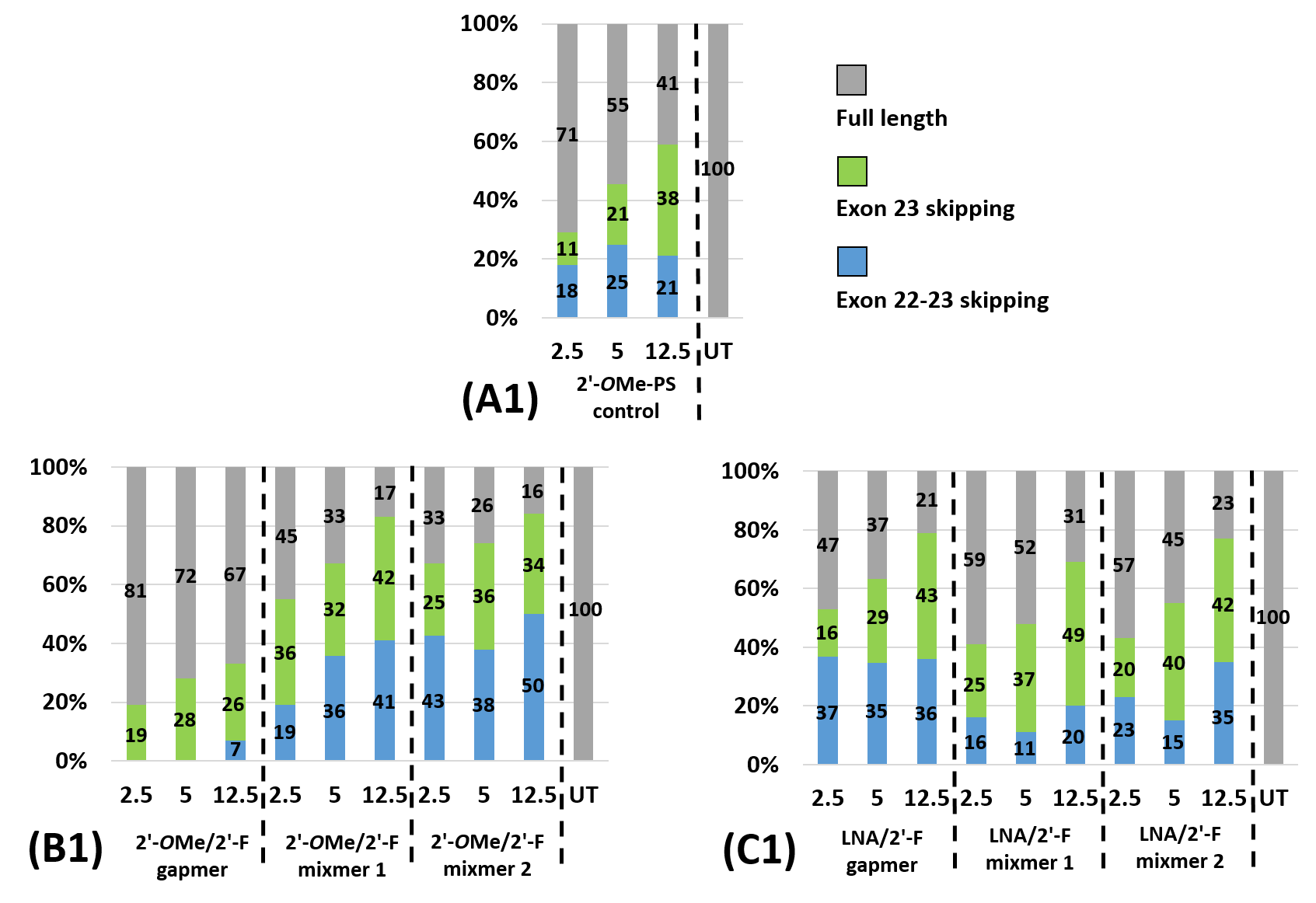


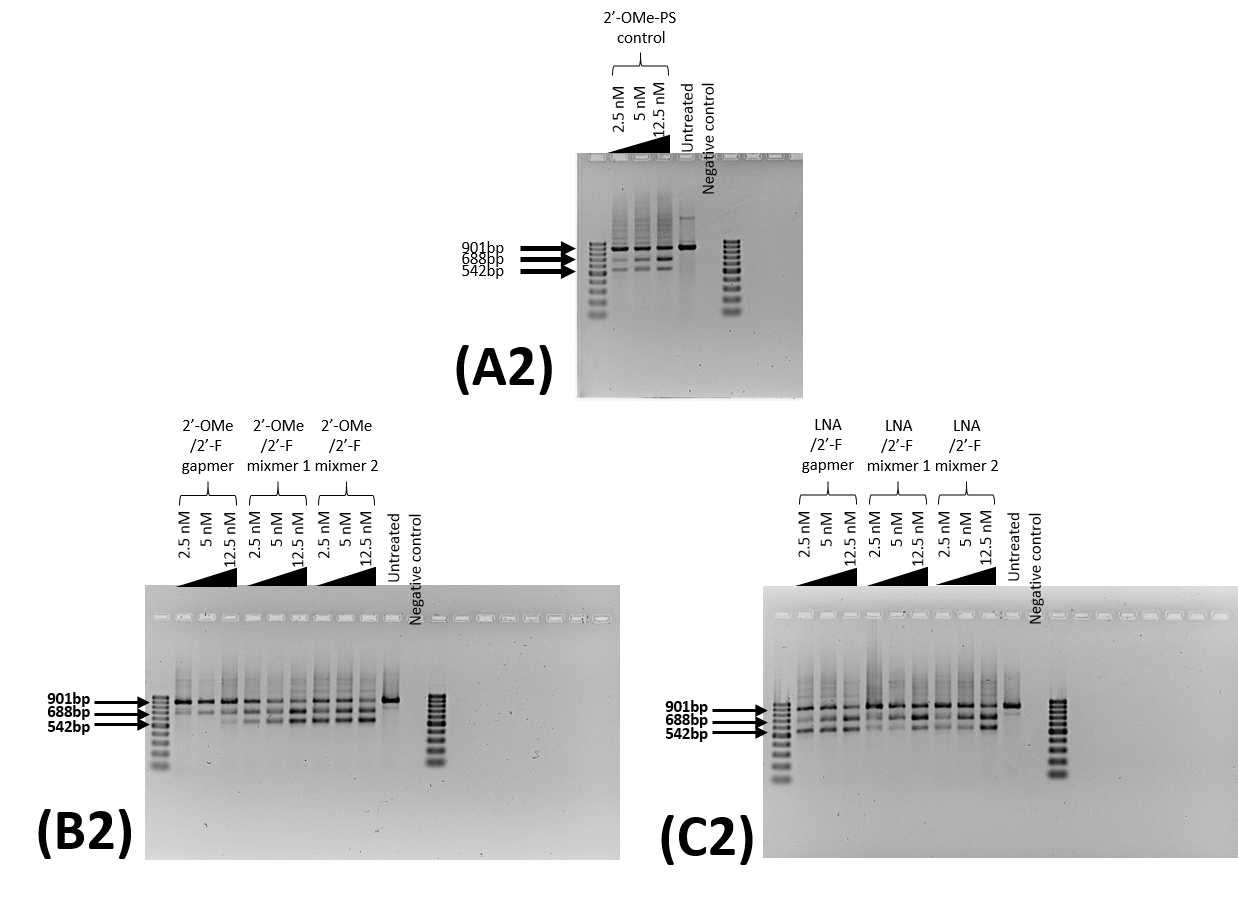


**Supplementary Figure S2**. Densitometry analysis and original gel images of repetition one of RT-PCR products showed exon-23 and exon-22/23 dual skipping in *mdx* mouse myotubes *in vitro*. Concentrations of AOs used include 2.5 nM, 5 nM, and 12.5 nM. (A1, A2) Fully modified 2'-*O*Me-PS control AO; (B1, B2) 2'-*O*Me modified 2'-F-PS AO chimeras including 2'-*O*Me/2'-F-PS gapmer, 2'-*O*Me/2'-F-PS mixmer 1, and 2'-*O*Me/2'-F-PS mixmer 2; (C1, C2) LNA modified 2'-F-PS AO chimeras including LNA/2'-F-PS gapmer, LNA/2'-F-PS mixmer 1, and LNA/2'-F-PS mixmer 2. [The original gels and densitometry data in this figure represent and/or support the gel images in Figure 4 and densitometry analysis in Figure 5 of the paper.]


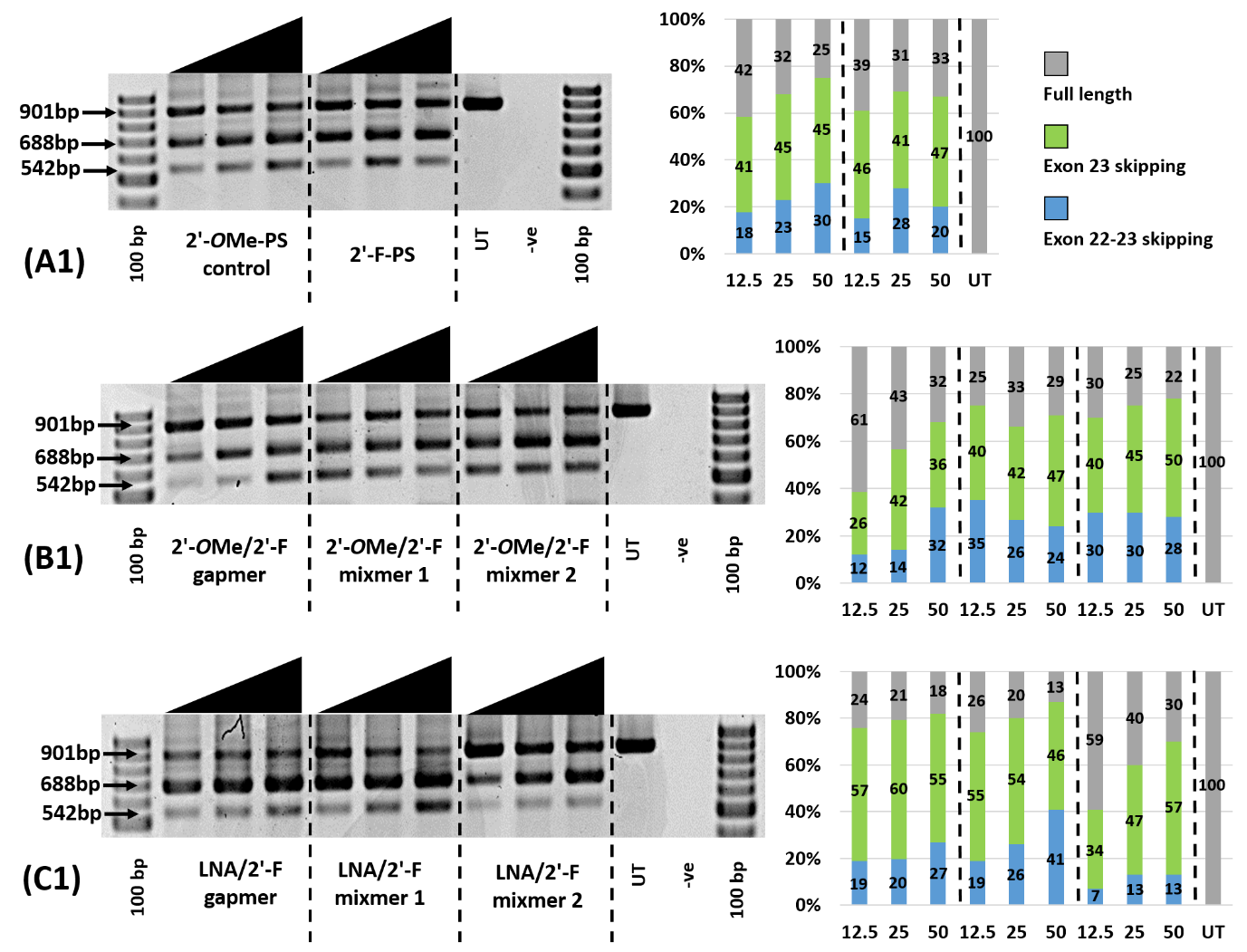


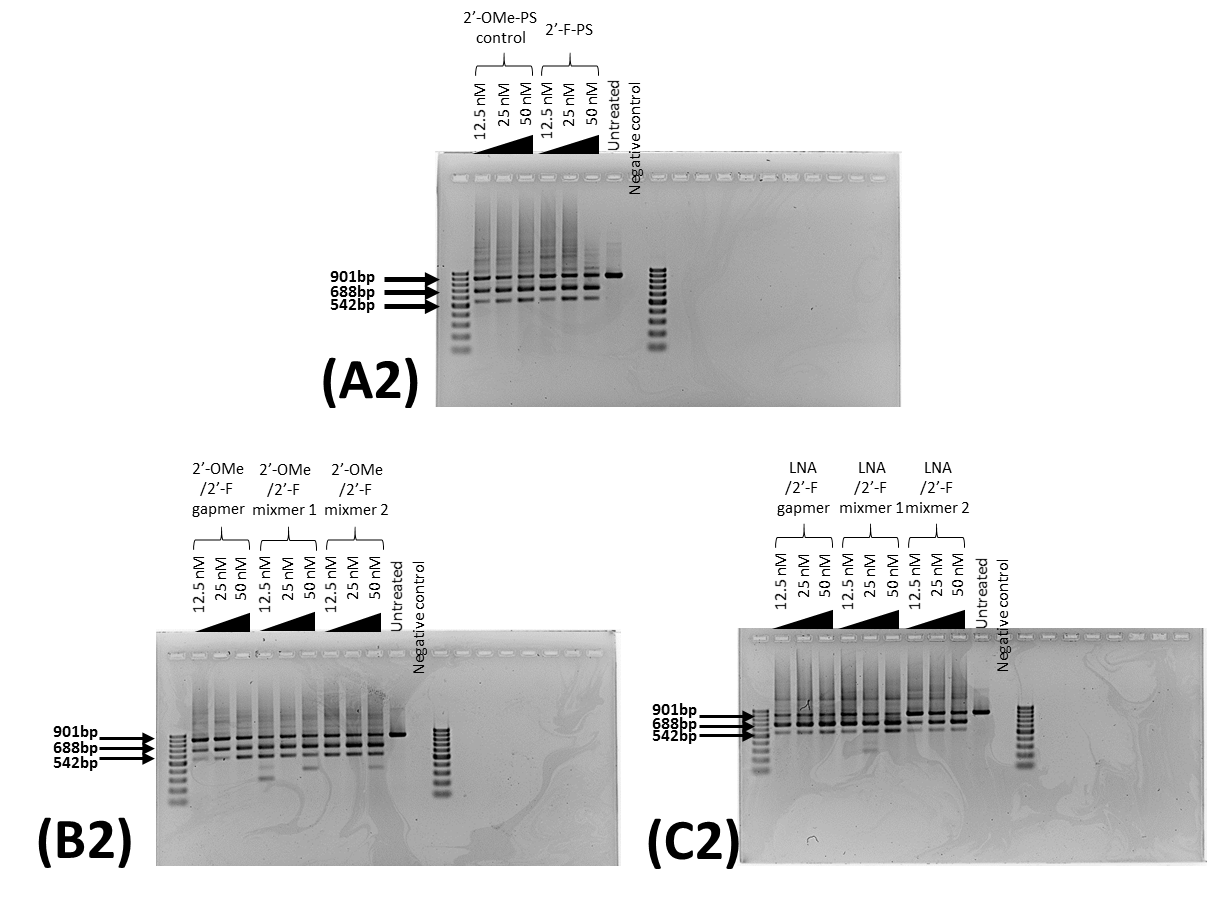


**Supplementary Figure S3**. Densitometry analysis and original gel images of repetition two of RT-PCR products showed exon-23 and dual exon-22/23 skipping in *mdx* mouse myotubes *in vitro*. Concentrations of AOs used include 12.5 nM, 25 nM, and 50 nM. (A1, A2) Fully modified 2'-*O*Me-PS control AO and fully modified 2'-F-PS AO; (B1, B2) 2'-*O*Me modified 2'-F-PS AO chimeras including 2'-*O*Me/2'-F-PS gapmer, 2'-*O*Me/2'-F-PS mixmer 1, and 2'-*O*Me/2'-F-PS mixmer 2; (C1, C2) LNA modified 2'-F-PS AO chimeras including LNA/2'-F-PS gapmer, LNA/2'-F-PS mixmer 1, and LNA/2'-F-PS mixmer 2. [The original gels and densitometry data in this figure represent and/or support the densitometry analysis in Figure 3 of the paper.]


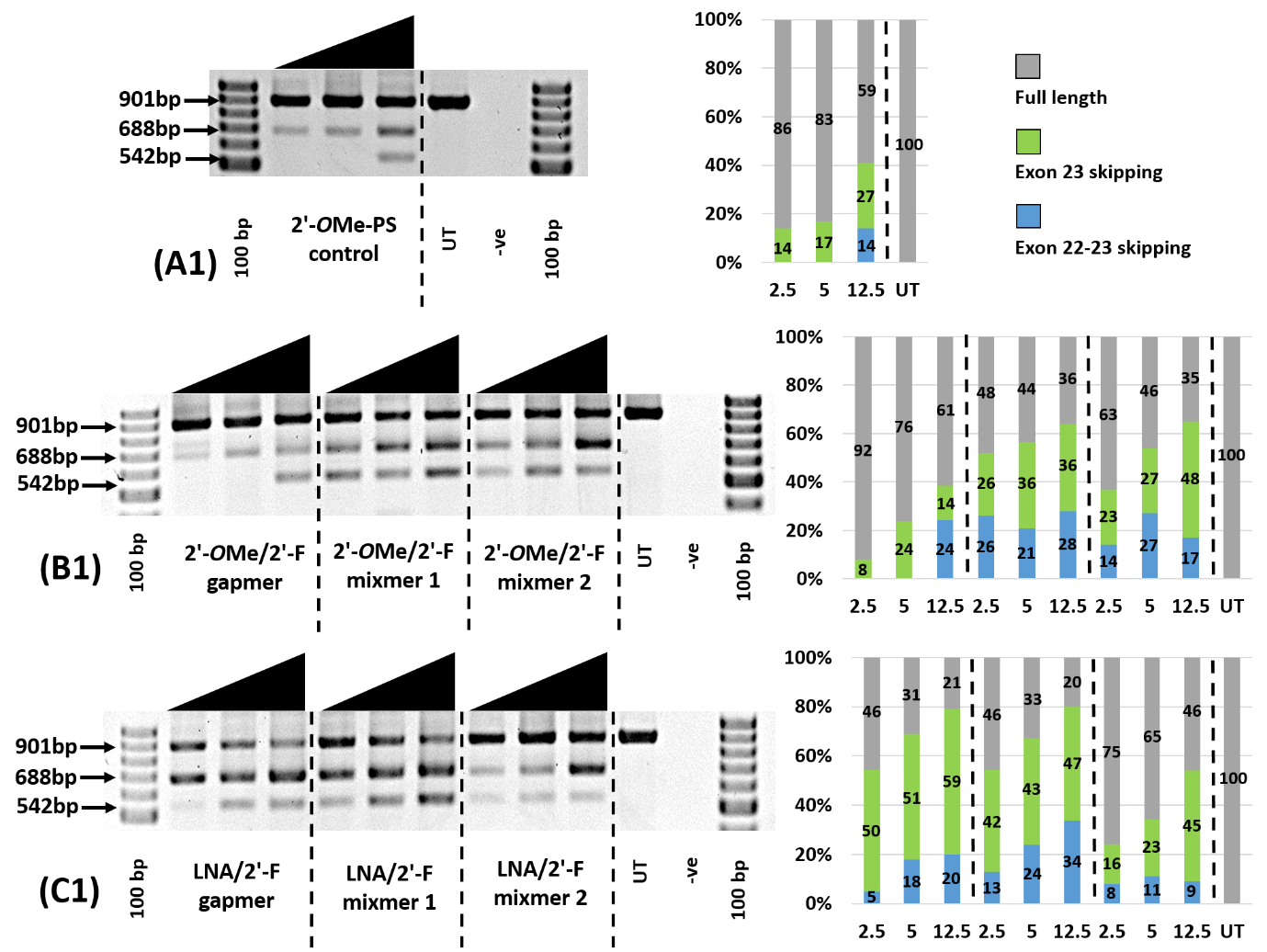


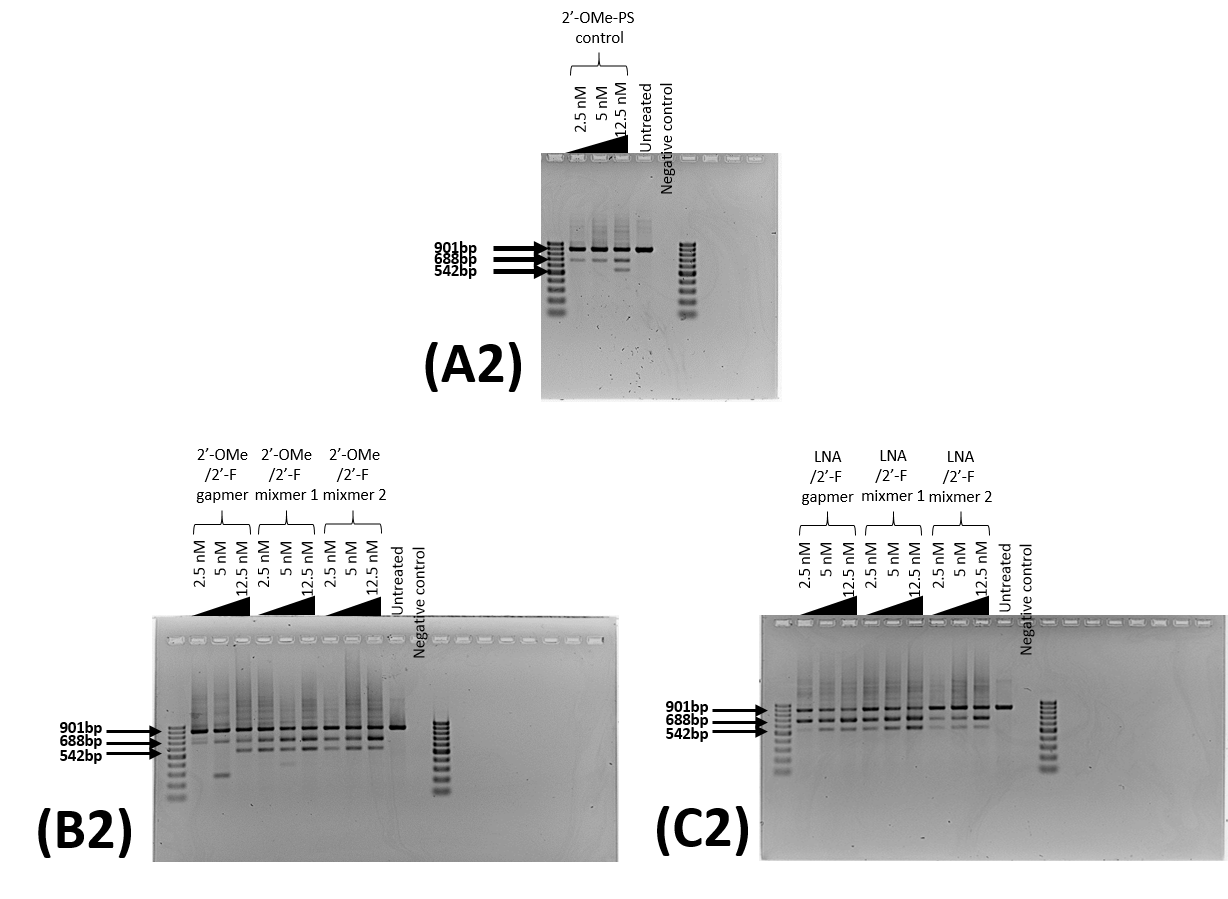


**Supplementary Figure S4**. Densitometry analysis and original gel images of repetition one of RT-PCR products showed exon-23 and exon-22/23 dual skipping in *mdx* mouse myotubes *in vitro*. Concentrations of AOs used include 2.5 nM, 5 nM, and 12.5 nM. (A1, A2) Fully modified 2'-*O*Me-PS control AO; (B1, B2) 2'-*O*Me modified 2'-F-PS AO chimeras including 2'-*O*Me/2'-F-PS gapmer, 2'-*O*Me/2'-F-PS mixmer 1, and 2'-*O*Me/2'-F-PS mixmer 2; (C1, C2) LNA modified 2'-F-PS AO chimeras including LNA/2'-F-PS gapmer, LNA/2'-F-PS mixmer 1, and LNA/2'-F-PS mixmer 2. [The original gels and densitometry data in this figure represent and/or support the densitometry analysis in Figure 5 of the paper.]

| Name of AOs | Concentration of AOs | | | | | |
| --- | --- | --- | --- | --- | --- | --- |
|  | 50 nM | | 12.5 nM | | 0 nM | |
|  | 1 | 2 | 1 | 2 | 1 | 2 |
| Untreated | - | - | - | - | 1.415 | 1.214 |
| 2'-*O*Me-PS control | 1.189 | 1.100 | 1.227 | 1.258 | - | - |
| 2'-F-PS | 1.115 | 1.222 | 1.336 | 1.253 | - | - |
| 2'-*O*Me/2'-F gapmer | 1.083 | 1.063 | 1.224 | 1.162 | - | - |
| 2'-*O*Me/2'-F mixmer 1 | 1.095 | 1.070 | 1.237 | 1.099 | - | - |
| 2'-*O*Me/2'-F mixmer 2 | 0.953 | 0.984 | 1.008 | 1.033 | - | - |
| LNA/2'-F gapmer | 1.065 | 1.067 | 1.191 | 1.300 | - | - |
| LNA/2'-F mixmer 1 | 1.139 | 1.147 | 1.188 | 1.283 | - | - |
| LNA/2'-F mixmer 2 | 1.137 | 1.156 | 1.327 | 1.233 | - | - |

**Supplementary Table S1**. The absorbance measured in a microplate reader (FLUOstar Omega, BMG Labtech) at 450 nm. [The data in this table is the original data representing Figure 6 (cell viability assay (in duplicates) of 2'-F modified AOs) of the paper.]


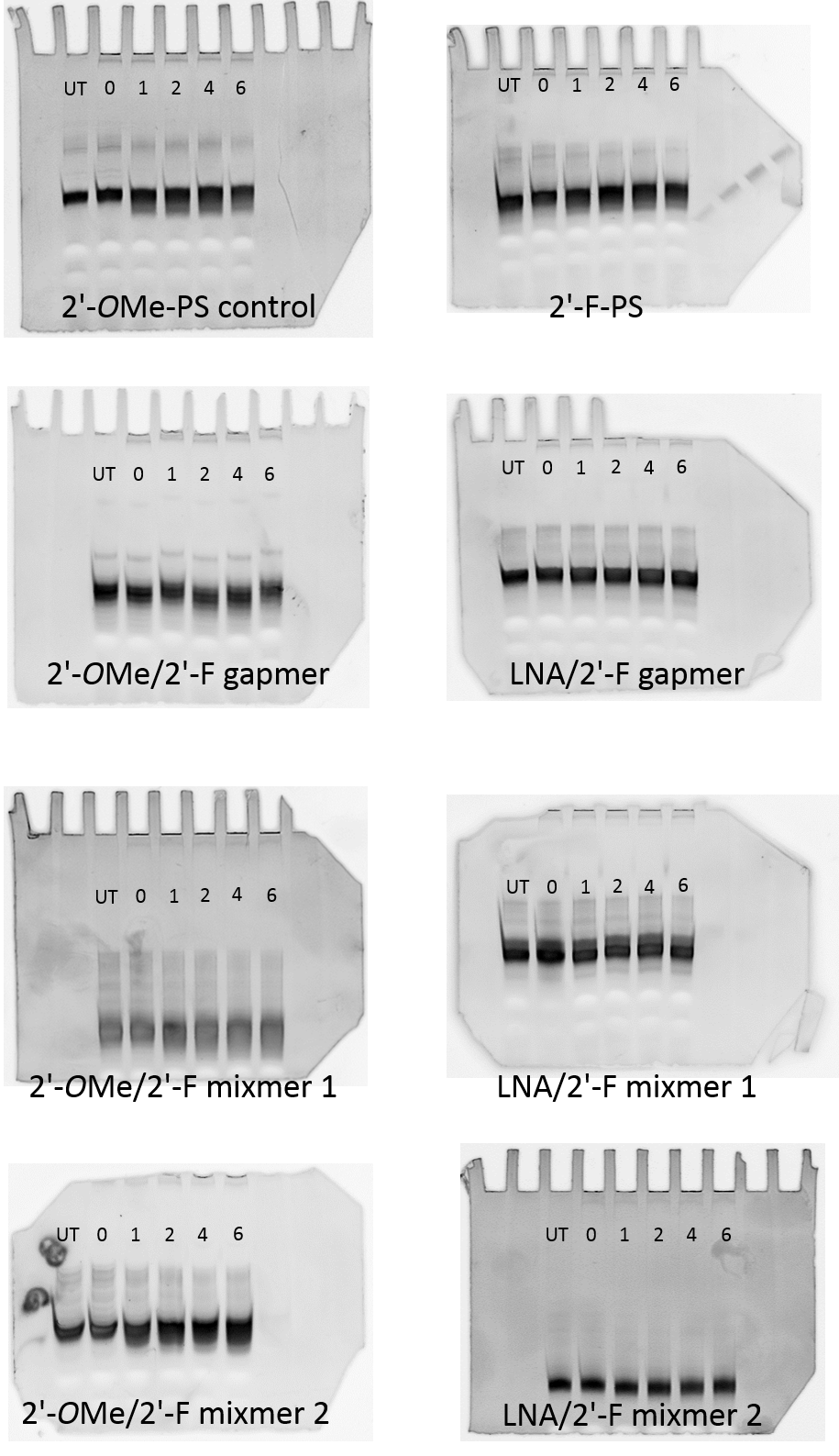


**Supplementary Figure S5**. Denaturing polyacrymide gel analysis of AOs. [The gels in this figure are the original gels representing Figure 7 (nuclease stability analysis of 2'-F modified AOs) of the paper.]
